# Early Life Exposure to Famine and Colorectal Cancer Risk: A Role for Epigenetic Mechanisms

**DOI:** 10.1371/journal.pone.0007951

**Published:** 2009-11-23

**Authors:** Laura A. E. Hughes, Piet A. van den Brandt, Adriaan P. de Bruïne, Kim A. D. Wouters, Sarah Hulsmans, Angela Spiertz, R. Alexandra Goldbohm, Anton F. P. M. de Goeij, James G. Herman, Matty P. Weijenberg, Manon van Engeland

**Affiliations:** 1 Department of Epidemiology, GROW School for Oncology and Developmental Biology, Maastricht University Medical Center, Maastricht, The Netherlands; 2 Department of Pathology, GROW School for Oncology and Developmental Biology, Maastricht University Medical Center, Maastricht, The Netherlands; 3 Department of Prevention and Health, TNO Quality of Life, Leiden, The Netherlands; 4 Sidney Kimmel Comprehensive Cancer Center, John Hopkins University School of Medicine, Baltimore, Maryland, United States of America; Lerner Research Institute, Cleveland Clinic, United States of America

## Abstract

**Background:**

Exposure to energy restriction during childhood and adolescence is associated with a lower risk of developing colorectal cancer (CRC). Epigenetic dysregulation during this critical period of growth and development may be a mechanism to explain such observations. Within the Netherlands Cohort Study on diet and cancer, we investigated the association between early life energy restriction and risk of subsequent CRC characterized by the (promoter) CpG island methylation phenotype (CIMP).

**Methodology/Principal Findings:**

Information on diet and risk factors was collected by baseline questionnaire (n = 120,856). Three indicators of exposure were assessed: place of residence during the Hunger Winter (1944–45) and World War II years (1940–44), and father's employment status during the Economic Depression (1932–40). Methylation specific PCR (MSP) on DNA from paraffin embedded tumor tissue was performed to determine CIMP status according to the Weisenberger markers. After 7.3 years of follow-up, 603 cases and 4631 sub-cohort members were available for analysis. Cox regression was used to calculate hazard ratios (HR) and 95% confidence intervals for CIMP+ (27.7%) and CIMP- (72.3%) tumors according to the three time periods of energy restriction, adjusted for age and gender. Individuals exposed to severe famine during the Hunger Winter had a decreased risk of developing a tumor characterized by CIMP compared to those not exposed (HR 0.65, 95%CI: 0.45–0.92). Further categorizing individuals by an index of ‘0–1’ ‘2–3’ or ‘4–7’ genes methylated in the promoter region suggested that exposure to the Hunger Winter was associated with the degree of promoter hypermethylation (‘0–1 genes methylated’ HR = 1.01, 95%CI:0.74–1.37; ‘2–3 genes methylated’ HR = 0.83, 95% CI:0.61–1.15; ‘4–7 genes methylated’ HR = 0.72, 95% CI:0.49–1.04). No associations were observed with respect to the Economic Depression and WWII years.

**Conclusions:**

This is the first study indicating that exposure to a severe, transient environmental condition during adolescence and young adulthood may result in persistent epigenetic changes that later influence CRC development.

## Introduction

It has long been hypothesized that the risk of adult disease is associated with dietary patterns experienced early in development [1]–[5]. Increasing evidence supports a role for epigenetic mechanisms in explaining how such early life exposures influence disease risk [6]–[10].

There are indications that severe, short-term energy restriction can induce persistent changes in epigenetic information. It was recently reported that individuals who were prenatally exposed to famine during the ‘Dutch Hunger Winter’ of 1944–45, had less DNA methylation of the imprinted IGF-2 gene 60 years later compared to their unexposed, same-sex sibling [6]. While this study did not look at specific adult disease as an endpoint, it showed for the first time that transient dietary patterns early in human gestation can induce permanent epigenetic changes. It also strengthened the hypothesis that likely, there are periods of temporal susceptibility to epigenetic modulations. Evidence suggests that windows of susceptibility also exist later in development [11]–[13], but this has never been investigated with respect to energy restriction.

In a large Dutch population, we have observed that individuals who experienced severe caloric restriction as adolescents through exposure to the Hunger Winter have a lower risk of developing colorectal cancer (CRC) later in life [14], [15]. CRC is one of the best described cancers in terms of genetic and epigenetic events involved [16]–[19]. A distinct characteristic of epigenetic instability in CRC is the CpG island methylator phenotype (CIMP), characterized by numerous promoter CpG island hypermethylated tumor suppressor- and DNA repair genes [20]–[24]. This in turn is associated with transcriptional silencing of gene expression [25]. Furthermore, many CIMP tumors also exhibit microsatellite instability (MSI)[26], a form of genetic instability associated with defective mismatch repair that is commonly achieved by promoter methylation of the mismatch repair gene *hMLH1* [16].

The Netherlands Cohort Study on diet and cancer (NLCS) offers a rare opportunity to study the association between energy restriction during childhood and adolescence and epigenetic changes associated with CRC. This large and well defined cohort is comprised of individuals who grew up during the Hunger Winter, as well as two preceding periods of more moderate energy restriction: World War II (1940–44), and the Economic Depression (1932–40). Although individual food intake data is not available for these three time periods, a number of proxy measures have been collected from cohort members that reflect their exposure to energy restriction. Additionally, colorectal tumor tissue samples have been collected from cases and subsequently analyzed for a wide range of molecular characteristics. Using this population, the present study investigated the association between energy restriction in childhood and adolescence and the risk of having a CIMP tumor later in life. Because CIMP tumors are associated with MSI, the relationship between energy restriction during childhood and adolescence and having a tumor characterized by MSI was also investigated.

## Methods

### Ethics Statement

The study protocol of the NLCS was approved by the Medical Ethics Committees of the University Hospital Maastricht and TNO Nutrition in February 1985 and July 1986, respectively. At the start of the study in 1986, participants were informed in writing of the details of the study and its objectives. In accordance with the regulations of the time, written informed consent was assumed when participants returned the completed baseline questionnaire. Paraffin embedded tumor material of the CRC patients was collected after approval by the ethical review boards of Maastricht University, the NCR (national cancer registry), and a national pathology database (PALGA) in 1997.

### Study Population and Design

The NLCS is a prospective cohort study that was initiated in 1986 with the purpose of investigating the association between diet and the development of cancer. The cohort included 58,279 men and 62,573 women between the ages of 55–69 years at baseline who completed a self-administered questionnaire on dietary habits, and lifestyle, health, and demographic information. Municipal registries from throughout the Netherlands were used to constitute an efficient sampling frame [27]–[29]. The NLCS uses a case-cohort approach for data processing and analysis; a subcohort of 5000 individuals was randomly selected from the larger cohort upon recruitment into the study. These individuals have been followed-up biennially from baseline in 1986 for migration and vital status to estimate person time at risk for the entire cohort. All subcohort members who reported prevalent cancer (excluding skin cancer) at baseline were excluded from analyses (leaving n = 4650). Further details of the design of the NLCS have been described in previous publications [27]–[29].

Incident CRC cases were identified by annual record linkage to nine regional cancer registries and PALGA. This method of record linkage has been described elsewhere [30]. The completeness of cancer follow-up has been estimated to be almost 100% [31]. CRCs were classified as proximal colon cancer (International Classification of Diseases for Oncology, first edition codes 153.0, 153.1, 153.4, 153.5 and 153.6), distal colon cancer (codes 153.2, 153.3 and 153.7), rectosigmoid (code 154.0) and rectal cancer (code 154.1). All prevalent cancer cases at baseline other than non-melanoma skin cancer and subjects with incomplete or inconsistent questionnaires were excluded.

Colorectal tumor samples were retrieved from August 1999 to December 2001, as described previously [32]. In total, 734 incident CRC patients were identified from a follow-up period of 7.3 years after baseline, of whom a PALGA report of the lesion as well as sufficient DNA was available [32].

### Assessment of Energy Restriction

Individual food intake data of the cohort members was not available for the three periods of energy restriction. Therefore, proxy variables were used to describe exposure to energy restriction.

With respect to the Economic Depression years, the best proxy available was the employment status of the cohort member's father during this time frame. It was documented that families with an unemployed father consumed fewer calories and had less variation in their food patterns than families with an employed father [14], [33]. On the baseline questionnaire, individuals were asked ‘was your father employed during the crisis of 1932–1940?’ Cohort members could either answer yes, along with the years that he was employed, or no. For analyses purposes, answers were categorized into ‘father was employed during the economic depression’ or ‘father was not employed during the economic depression’.

For the years spanning both WWII and the Hunger Winter, place of residence was used to approximate the exposure to energy restriction. During WWII, food rationing was introduced and because of poor food availability in large cities, nutritional differences emerged between people living in urban versus rural areas. It was documented that individuals living in a city with more than 40,000 inhabitants consumed fewer calories daily, and ate diets consisting of a higher percentage of carbohydrates (70% vs 65%) and a lower percentage of fat (10% vs 15%) [14], [34]. Therefore, place of residence in 1942 (midpoint of the war years) was used as a proxy for energy restriction during WWII. On the baseline questionnaire, cohort members were asked to list the last four residences they lived in, the province where these residences were located, and the years that they lived in these locations. The location of residence during the mid-point of WWII was dichotomized to ‘lived in a city in 1942’, or ‘lived in a rural area in 1942’.

Additionally, cohort members were specifically asked where they lived during the winter of 1944–45. Residing in a Western city during the Hunger Winter was considered an indicator for severe energy restriction. While the diet remained nutritionally balanced, individuals living in western urban areas experienced rationing of less than 700 kcal per day [35], [36]. Eleven Western cities were considered famine cities based on the definition by Stein et al [37]: Amsterdam, Rotterdam, The Hague, Utrecht, Zaandam, Hilversum, Amersfoort, Dordrecht, Vlaardingen/Schiedam, Delft, and Leiden. Answers to this question were classified into three categories: lived in a Western city, lived in a Western rural area, and lived in a non-Western area of the Netherlands during the winter of 1944–1945.

### Promoter Methylation Analyses

CIMP in tumor tissue of CRC cases was defined by CpG island promoter hypermethylation of at least 3 out of 5 methylation markers (*CACNA1G, IGF2, NEUROG1, RUNX3* and *SOCS1*), as proposed by Weisenberger et al [23]. Methylation status of these markers, as well as that of the *MLH1* and *APC* gene; used to create a methylation index with the CIMP markers, were determined by bisulfite modification of 500 ng genomic DNA using a commercially available kit (Zymo Research), and subsequent methylation specific PCR (MSP) [38], [39]. We chose to use MSP as a method because it is effective, specific and does not require specific equipment. It has been shown that results from MSP are in accordance with other technologies, such as MethyLight [24]. To facilitate MSP analysis on DNA retrieved from formalin-fixed, paraffin-embedded tissue, DNA was first amplified with flanking PCR primers that amplify bisulfite-modified DNA but do not preferentially amplify methylated or unmethylated DNA. The resulting fragment was used as a template for the MSP reaction. All PCRs were carried out with controls for unmethylated alleles (DNA from normal lymphocytes), methylated alleles [normal lymphocyte DNA treated *in vitro* with *Sss*I methyltransferase (New England Biolabs, lpswich, MA)] and a control without DNA. Ten microliters of each MSP reaction was directly loaded on to nondenaturing 6% polyacrylamide gels, stained with ethidium bromide and visualised under UV illumination. The MSP analyses were successful for 81%, 79%, 79%, 90%, 83%, 93%, and 93% out of the 734 cases for *CACNA1G, IGF2, NEUROG1, RUNX3, SOCS1*, *MLH1*, and *APC* respectively.

### Microsatellite Instability Analyses

MSI was determined by a pentaplex PCR using the MSI markers BAT-26, BAT-25, NR-21, NR-22 and NR-24, as described in detail by Suraweera et al [40]. MSI analyses were successful on 662 (90%) of the 734 available DNA samples.

### Data Analyses

Data were analyzed with Stata (version 9.2, Statacorp, College Station, TX, USA). Cox proportional hazards analysis using the case-cohort approach was used to obtain hazard ratios (HR) and 95% confidence intervals (CI) for the association between the three proxy measures of energy restriction and CRC characterized by CIMP and MSI status. The proportional hazards assumption was tested using the scaled Schoenfeld residuals and visual inspection of the hazard curves. To account for the additional variance introduced by sampling the subcohort from the entire cohort, standard errors were estimated using the robust option.

Potential confounding variables considered for multivariate analysis were age, sex, BMI (kg/m^2^), height (cm), family history of CRC (yes/no), smoking status (never smoker, ex-smoker, current smoker), level of education (primary school, junior high school, senior high school, higher vocational school, or university), total energy intake (kcal/day), alcohol intake (g/day), recreational physical activity (<30 minutes/day, 30–60 minutes/day, 61–90 minutes/day, >90 minutes/day), and consumption of red meat, fruit, vegetables, and grains (g/day). For women, hormonal factors such as contraceptive use (yes/no), age at menarche, age at menopause, and number of children were also considered. Those variables that changed the risk estimates for the association of energy restriction and CRC by more than 10% were ultimately included as confounders. After adjustment for age and sex, effect estimates remained extremely stable. HRs were therefore estimated using a model that only included age and sex, and expressed using ‘father was employed during the economic depression’, ‘lived in a rural area in 1942’, and ‘lived in a non-western area of the Netherlands during the Hunger Winter’ as the reference categories for the different proxies of energy restriction.

To assess how exposure to energy restriction was associated with the extent of promoter methylation in the CRC tumors, a methylation index was created that consisted of the five CIMP markers (*CACNA1G, IGF2, NEUROG1, RUNX3, and SOCS1)*, and the *MLH1* and *APC* genes. Cases were categorized into one of three groups: ‘0–1 genes methylated’, ‘2–3 genes methylated’, or ‘4–7 genes methylated’. Of the 734 cases, 556 had sufficient information to be classified into one of the three categories.

Tests for heterogeneity were done to evaluate differences between subtypes of tumors (e.g., *CIMP vs. non-CIMP*) using the competing risks procedure in STATA. However, the SE for the difference of the log–hazard ratios from this procedure assumes independence of both estimated hazard ratios, which would overestimate that SE and thus overestimate the *P* values for their difference. Therefore, these *P* values and the associated confidence intervals were estimated based on a bootstrapping method that was developed for the case-cohort design [41]. For each bootstrap sample, *X* subcohort members were randomly drawn from the subcohort of *X* subjects and *Y* cases from the total of *Y* cases outside the subcohort, both with replacement, out of the data set of *X*+*Y* observations. The log–hazard ratios were obtained from this sample using STATA's competing risks procedure and recalculated for each bootstrap replication. The confidence interval and *P* value of the differences in hazard ratio of the subtypes were then calculated from the replicated statistics using the accelerated bias corrected method in STATA. Each bootstrap analysis was based on 1,000 replications [42].

## Results

The distribution of exposure variables for the study population is presented in table 1.

**Table 1:** Distributions of energy restriction exposure variables for CRC cases characterized by CIMP*, MSI, and sub-cohort members in the Netherlands Cohort Study on diet and cancer after 7.3 years of follow-up (1986-1993).

		CRC cases characterized by:			
	sub-cohort (n = 4650)†	CIMP+ (n = 167)†	CIMP- (n = 436)†	MSI+ (n = 84)†	MSI- (n = 578)†
**Exposure variables Hunger Winter 1944-45** ¶					
*Non-Western*	2369 (58.3)‡	100 (68.5)§	224 (60.9)§	46 (62.2)§	304 (62.5)§
*Western Rural*	626 (15.4)	13 (8.9)	49 (13.3)	9 (12.2)	64 (13.7)
*Western City*	1067 (26.3)	33 (22.6)	95 (25.8)	19 (25.7)	118 (24.3)
**War Years 1940-44** ¶					
*Rural Area in 1942*	1648 (48.8)	58 (47.9)	179 (52.5)	32 (49.2)	233 (52.7)
*Urban area in 1942*	1732 (51.2)	63 (52.1)	162 (47.5)	33 (50.8)	209 (47.3)
**Economic Depression 1932-40** ¶					
*Father had a job*	3864 (88.3)	142 (88.1)	371 (90.3)	75 (92.6)	485 (89.3)
*Father did not have a job*	508 (11.6)	19 (11.8)	40 (9.7)	6 (7.4)	58 (10.7)

* CIMP status defined as ≥3/5 genes methylated according to the Weisenberger markers

(CACNA1G, IGF2, NEUROG1, RUNX3, and SOCS1).

† due to missing data for exposure variables, numbers may not add up to 4650, 167, 84 and 578, respectively.

‡ presented as number of sub-cohort members (% of total number of sub-cohort members in the exposure category).

§ presented as number of cases (% of total number of cases in the exposure category).

¶ *Hunger Winter:* it was assumed that individuals residing in a western area of the Netherlands, especially a western urban area during the Hunger Winter would have experienced the greatest degree of energy restriction; *War Years:* it was assumed that individuals who resided in an urban area during the mid-point of the War would have experienced the greatest degree of energy restriction; *Economic Depression*: it was assumed that individuals who had an unemployed father during the Economic Depression would have experienced a higher degree of energy restriction compared to those whose father was employed.

For all of the case groups, there were proportionally more individuals residing in a non-western area during the Hunger Winter compared to subcohort members. Proportionally, there were fewer non-CIMP and non-MSI cases residing in an urban area during WWII than rural area than CIMP+ cases, MSI+ cases, and sub-cohort members. With respect to the Economic Depression, more MSI+ cases had an employed father compared to the other case groups, and sub-cohort members.

Associations between the three proxy measures of energy restriction and risk of tumors with specific characteristics are presented in table 2. Individuals who reported living in a western area of the Netherlands during the Hunger Winter had a statistically significant lower risk of developing a CIMP tumor compared to those who did not (HR =  0.65, 95% CI: 0.45–0.92). Inverse associations remained when considering ‘western area’ as either western rural area (HR =  0.51, 95% CI: 0.28–0.91), or western city (HR = 0.72, 95%CI: 0.48–1.08). There was no association between exposure to energy restriction during WWII and the Economic Depression and having a CIMP tumor. Tests for heterogeneity showed no significant difference between the HRs observed for tumors with and without CIMP. Inverse associations were observed with respect to the three time periods of energy restriction and risk of a tumor with MSI, but these were far from statistically significant. Tests for heterogeneity showed no significant difference between the HRs observed for tumors with and without MSI.

**Table 2:** Age and sex adjusted and hazard rate ratios (HR) for CRC characterized by CIMP and MSI, according to three time periods of energy restriction in the Netherlands Cohort Study on diet and cancer (7.3 years of follow-up).

	Hunger Winter (1944–45)‡				War Years (1940–44) ‡		Economic Depression(1932–40)‡	
	*Non-western* *	*Western rural*	*Western city*	*Western areas combined* †	*Rural area in 1942* *	*Urban area in 1942*	*Father had a job* *	*Father did not have a job*
**Person years at risk**	16,894	4483	7630	12,133	11,737	12,391	27,575	3608
**CRC endpoints characterized by:**								
**CIMP**								
*Number of cases*	100	13	33	46	58	63	142	19
*HR (95% CI)*	1.00	0.51 (0.28–0.91)	0.72 (0.48–1.08)	0.65 (0.45–0.92)	1.00	0.99 (0.69–1.43)	1.00	0.98 (0.61–1.60)
**Non-CIMP**								
*Number of cases*	224	49	95	114	179	162	371	40
*HR (95% CI)*	1.00	0.85 (0.62–1.18)	0.94 (0.73–1.21)	0.91 (0.73–1.23)	1.00	0.84 (0.67–1.05)	1.00	0.81 (0.57–1.13)
**MSI**								
*Number of cases*	46	9	19	28	32	33	75	6
*HR (95% CI)*	1.00	0.77 (0.37–1.59)	0.90 (0.52–1.54)	0.85 (0.53–1.37)	1.00	0.94 (0.57–1.54)	1.00	0.58 (0.25–1.34)
**Non-MSI**								
*Number of cases*	304	64	118	182	233	209	485	58
*HR (95% CI)*	1.00	0.82 (0.62–1.09)	0.86 (0.68–1.08)	0.84 (0.69–1.03)	1.00	0.83 (0.68–1.01)	1.00	0.89 (0.66–1.19)

* reference category.

† category created from combining individuals who lived in a western rural area and a western urban area.

*‡ Hunger Winter:* it was assumed that individuals residing in a western area of the Netherlands, especially a western urban area during the Hunger Winter would have experienced the greatest degree of energy restriction; *War Years:* it was assumed that individuals who resided in an urban area during the mid-point of the War would have experienced the greatest degree of energy restriction; *Economic Depression*: it was assumed that individuals who had an unemployed father during the Economic Depression would have experienced a higher degree of energy restriction compared to those whose father was employed.

HRs for the three proxies of energy restriction according to a methylation index of 7 genes are presented in table 3. Of the 556 cases with sufficient information to be categorized, 37% were identified as having 0–1 genes methylated, 37% as having 2–3 genes methylated, and 26% as having > 4–7 genes methylated. Categorizing individuals by ‘0–1 genes methylated’, ‘2–3 genes methylated’ and ‘4–7 genes methylated’, suggested that residing in a western area during the Hunger Winter was associated with the degree of promoter hypermethylation (‘0–1 genes methylated’ HR = 1.01, 95%CI: 0.74–1.37; ‘2–3 genes methylated’ HR = 0.83, 95% CI: 0.61–1.15; ‘4–7 genes methylated’ HR = 0.72, 95% CI: 0.49–1.04) compared to individuals who did not reside in a western area.

**Table 3:** Age and sex adjusted hazard rate ratios (HR) for a methylation index according to three time periods of energy restriction in the Netherlands Cohort Study on diet and cancer (7.3 years of follow-up).

	Hunger Winter (1944–45)§				War Years (1940–44)§		Economic Depression(1932–40)§	
	*Non-western* *	*Western rural*	*Western city*	*Western areas combined* †	*Rural area in 1942* *	*Urban area in 1942*	*Father had a job* *	*Father did not have a job*
**Person years at risk**	16,894	4483	7630	12,133	12,391	11,737	27,575	3608
**Number of genes methylated** ‡ **:**								
**0–1**								
*Number of cases*	102	24	49	73	82	85	171	20
*HR (95% CI)*	1.00	0.92 (0.58–1.46)	1.06 (0.74–1.50)	1.01 (0.74–1.37)	1.00	0.95 (0.69–1.30)	1.00	0.86 (0.54–1.39)
**2–3**								
*Number of cases*	107	24	39	63	90	66	180	18
*HR (95% CI)*	1.00	0.87 (0.55–1.37)	0.82 (0.56–1.19)	0.84 (0.61–1.15)	1.00	0.68 (0.49–0.94)	1.00	0.75 (0.46–1.24)
**4–7**								
*Number of cases*	84	11	32	43	49	55	122	15
*HR (95% CI)*	1.00	0.51 (0.27–1.30)	0.83 (0.55–1.26)	0.72 (0.49–1.04)	1.00	1.02 (0.68–1.52)	1.00	0.90 (0.52–1.56)

* reference category.

† category created from adding together individuals who lived in a western rural area and a western urban area.

‡ methylation index including the five CIMP markers (*CACNA1G, IGF2, NEUROG1, RUNX3, and SOCS1)*, *MLH1,* and the *APC* gene.

*§ Hunger Winter:* it was assumed that individuals residing in a western area of the Netherlands, especially a western urban area during the Hunger Winter would have experienced the greatest degree of energy restriction; *War Years:* it was assumed that individuals who resided in an urban area during the mid-point of the War would have experienced the greatest degree of energy restriction; *Economic Depression*: it was assumed that individuals who had an unemployed father during the Economic Depression would have experienced a higher degree of energy restriction compared to those whose father was employed.

## Discussion

To our knowledge, this study is the first to show that energy restriction during adolescence and early adulthood is associated with the CIMP phenotype in CRC, suggesting that exposure to a transient environmental condition during this period of life may result in persistent epigenetic changes that later influence CRC development. We observed that individuals exposed to the Hunger Winter of 1944–45, a period of severe short-term energy restriction, had a decreased risk of developing a CIMP tumor later in life compared to individuals who were not exposed. Additionally, considering a methylation index suggested that the degree of hypermethylation was inversely associated with exposure to energy restriction. These observations, along with the fact that there was no association between exposure and risk of MSI, suggest that energy restriction during childhood and adolescence is associated with CIMP risk independently of MSI. Furthermore, the strong associations we observed during the period of the Hunger Winter suggest that it may be severe caloric restriction as opposed to specific nutrient restriction that modulates the risk of colorectal tumors with the CIMP phenotype, as we observed no associations during the other two periods of exposure when caloric restriction was assumed to be less severe.

Before discussing our results further, it is important to address some important limitations in our study. Although the number of CRC cases after 7.3 years of follow up in the NLCS was substantial, the number of these cases with the CIMP and or MSI phenotype was small. Because information on individual food for the cohort was not available for the exposure periods, three proxy measures of energy restriction were used to describe energy restriction during the Economic Depression, WWII, and the Hunger Winter. It is possible that using the father's employment status, place of residence during WWII, and place of residence during the Hunger Winter has resulted in some misclassification of exposure; however, in a follow-up study in the NLCS, female sub-cohort members were asked if they really had experienced hunger during the winter of 1944–1945. Of the women who reported severe hunger, 80% lived in a western city during this winter [43]. These results indicate that place of residence during the Hunger Winter is a reasonably adequate proxy of exposure, although unavoidable misclassification may have caused attenuation of the association. While it has been proposed that individual famine data may be more accurate than area-exposure data [44], other studies in the Netherlands have used place of residence during the Hunger Winter as a measure of energy restriction [45], [46] and their results indicate that it is an adequate proxy.

Although there is thought that environmental influences on epigenetics are likely to be most important during prenatal and early postnatal development, when epigenetic mechanisms undergo establishment and maturation [9], our findings strengthen the hypothesis that childhood and adolescence may also be periods of temporal susceptibility to epigenetic modulations [12], [13]. Perhaps, this is especially true for tissue specific changes, such as in the colorectal tract where cells are constantly being regenerated. The epigenetic process involved in normal gastrointestinal development, including the expression of gut enzymes, hormones, transporters, and the maturation of gastrointestinal immunoregulation, is an understudied area of research [7]. Therefore, we can only speculate how an environmental exposure, such as energy restriction, during childhood and adolescence might induce persistent epigenetic changes that later influence methylation patterns in CRC.

Waterland et al. propose that environmental influences on the developmental establishment of DNA methylation can occur through two mechanisms: by affecting the supply of dietary methyl donors and/or activity of DNA methyltransferases to induce either hyper- or hypomethylation at metastable epialleles, or by altering transcriptional activity of specific genes during ontogenic periods when DNA methylation is being established [9]. The pre-pubertal and pubertal years are a period of rapid growth and hormonal change, and there could be a link between the influx of growth hormones and regulation of epigenetic mechanisms controlling cancer causing genes. Growth hormone affects cellular growth through the actions of insulin growth factors, and it has been suggested that the beneficial effect of caloric restriction on CRC risk is mediated through decreasing IGF-1 [47]. During puberty, IGF-1 can be as high as four times the normal adult serum concentration [47]. It could be possible that energy restriction during this critical period of growth may permanently influence the growth hormone-IGF axis, subsequently influencing methylation patterns later in life. Alternatively, imprinted genes, which are vital for human development, may be involved in modulating future risk. Loss of imprinting of the IGF-2 gene is an example of epigenetic dysregulation in gastrointestinal carcinogenesis [7]. While it has been reported that adult diet does not correlate with IGF-2 loss of imprinting, a study in mice has shown that genomic imprinting may be modifiable through altering the diet in the postnatal period [48]. It would be interesting to determine if genomic imprinting is also modifiable during childhood and adolescence.

Our observations provide insight into how energy restriction early in life may influence the development of colorectal cancer [15], [49]–[52], and also generate hypotheses for future research. In contrast to the nutritional circumstances of the Netherlands during the mid-20^th^ century, most Western countries now find themselves in a situation of having too much, rather than too little. It has been observed that being overweight during adolescence may be a more powerful predictor of CRC risk than being overweight during adulthood [53]. Determining if energy excess during childhood and adolescence influences epigenetic mechanisms in a way that increases future disease risk is important to elucidate; both in developed countries where the prevalence of childhood obesity is reaching epidemic proportions, and in developing countries where transitioning nutritional habits and exposures may have implications on future CRC trends.

In conclusion, while a dark moment in Dutch history, unique evidence from the Hunger Winter shows that exposure to a period of severe transient energy restriction during adolescence is inversely associated with the risk of having a CIMP tumor later in life. These findings suggest that adolescence may be a critical period of development for epigenetic changes that influence CRC risk.
